# Diagnosis of Anomalous Origin of the Left Coronary Artery from the Pulmonary Artery with Echocardiography and Digital Subtraction Angiography

**DOI:** 10.1155/2018/5728782

**Published:** 2018-02-15

**Authors:** Haiyan Yang, Jinqing Li, Xiaojuan Ji

**Affiliations:** ^1^Department of Cardiology, Children's Hospital of Chongqing Medical University, Chongqing 400014, China; ^2^Ministry of Education Key Laboratory of Child Development and Disorders, Chongqing Key Laboratory of Child Infection and Immunity, China International Science and Technology Cooperation Base of Child Development and Critical Disorders, Chongqing 400014, China; ^3^Department of Radiology, 324th Hospital of the PLA, Chongqing 400020, China

## Abstract

Anomalous origin of the left coronary artery from the pulmonary artery (ALCAPA) is a common coronary artery anomaly associated with high mortality and may lead to sudden death if left unrecognized and untreated. This report describes an 8-year-old female who had cardiac murmur but with no clinical symptoms. Electrocardiogram (ECG) was normal, but echocardiography made the diagnosis of ALCAPA. Digital subtraction angiography (DSA) with cardiac catheterization angiography (CAG) confirmed the diagnosis, and finally, the patient received surgery. This case demonstrates that echocardiography is a sensitive and convenient technique for establishing the initial diagnosis of ALCAPA in both symptomatic and asymptomatic patients.

## 1. Introduction

ALCAPA is a rare congenital cardiovascular malformation in which the left coronary artery originates from the pulmonary artery (PA) instead of the left coronary sinus [1]. The incidence is 1 in 300,000 live births and accounts for 0.25% to 0.5% of congenital heart disease cases. ALCAPA results in mortality as high as 90% in the first year of life if the patient is not treated in a timely fashion [2]. Here, we present a case of a female patient diagnosed with ALCAPA. The patient's medical records, such as clinical symptoms, electrocardiogram (ECG), echocardiography, and cardiac catheterization angiography (CAG) images, were analyzed retrospectively.

## 2. Case Report

An 8-year-old Chinese female with a cardiac murmur was initially diagnosed as having a ventricular septal defect (VSD) in the local hospital. She was asymptomatic, had no difficulty breathing, and displayed neither increased diaphoresis nor cyanosis, but she developed upper respiratory infections more often than her peers and had reduced exercise capacity compared to her peers. On physical examination, she had the following vital measurements: 24 kg weight, 121 cm height, blood pressure 115/51 mm·Hg, heart rate 92/min, respiratory rate of 22/min, and transcutaneous oxygen saturation (SpO_2_) 96%. The results of physical examination were normal when compared with the same age of females within same race. On cardiac examination, her pulse and S1 and S2 were normal, but she had a grade 2/6 holosystolic murmur at the second left intercostal space.

**Figure 1:**
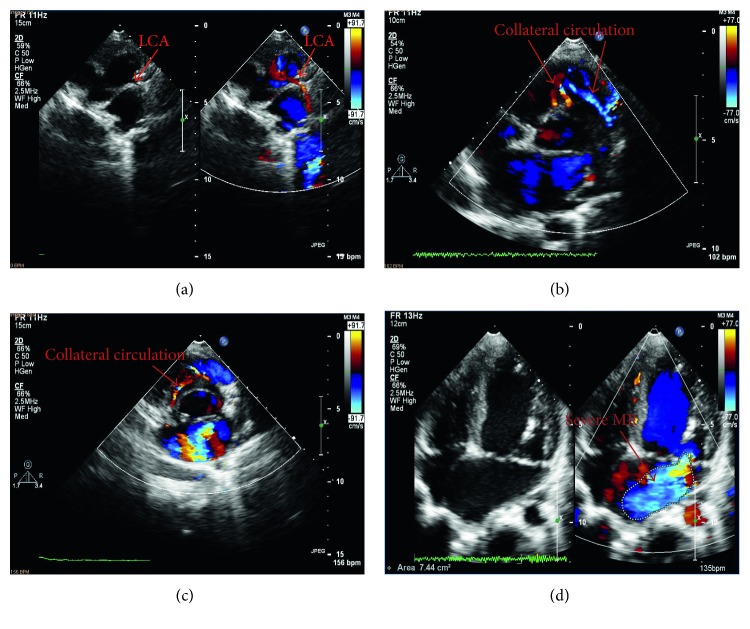
(a) The left coronary artery (LCA) originating from the pulmonary artery. (b) Reversal of blood flow in the left coronary artery (LCA) and abundant collateral circulation. (c) The abundant collateral circulation. (d) Severe mitral regurgitation (MR).

ECG displayed normal sinus rhythm and no signs of myocardial ischemia. Chest X-ray revealed an enlarged cardiac silhouette. Echocardiography demonstrated ALCAPA, right coronary artery to right ventriclar fistula, right coronary artery aneurysm, collateral coronary circulation formation, severe mitral regurgitation (MR), left ventricular ejection fraction (LVEF) of 61%, and left ventricular fractional shortening (LVFS) of 33% (Figure 1).

**Figure 2:**
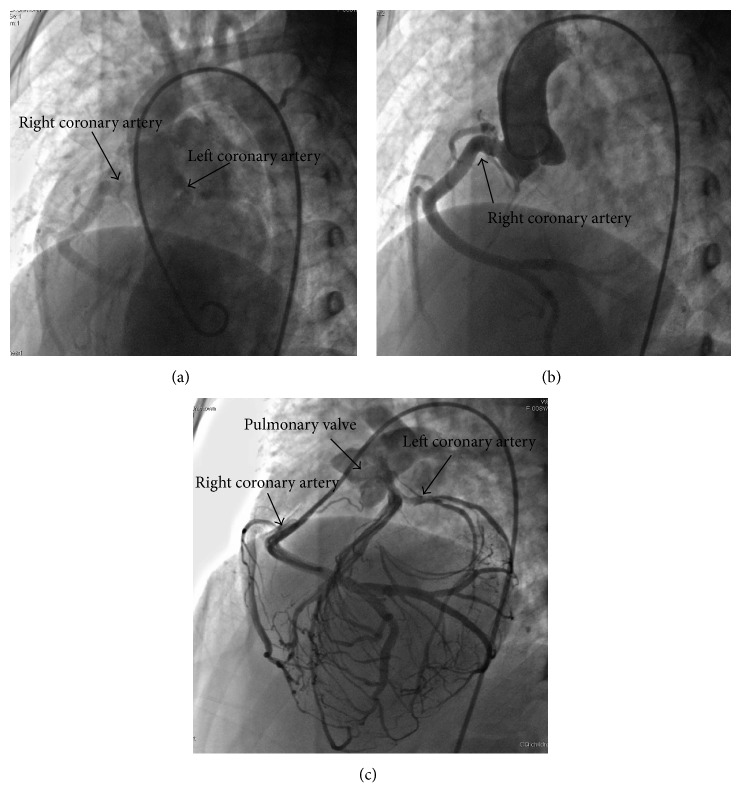
(a) Left ventricle angiography visualizing both right coronary artery (RCA) and delayed filling of the left coronary artery (LCA). (b) The aortic root angiography; only right coronary artery (RCA) originating from the right coronary cusp was imaged immediately. (c) Pulmonary arterial angiography; the left coronary artery (LCA) was imaged immediately, originating from the pulmonary artery; the RCA was then imaged via abundant collateral circulation.

Subsequently, cardiac catheterization and left ventricular angiography demonstrated a dilated right coronary artery (RCA) and with delayed filling of a dilated left coronary artery (LCA). There was no shunt across the interventricular septum. Then, aortic root angiography showed the dilated RCA originating from the right coronary cusp (RCC), and delayed enhanced-imaging demonstrated the LCA filling via extensive intercoronary collateral circulation. Finally, PA angiography showed immediate filling of the LCA, which originated from the PA; then abundant collateral circulation and delayed RCA were imaged (Figure 2), confirming the diagnosis of ALCAPA.

**Figure 3:**
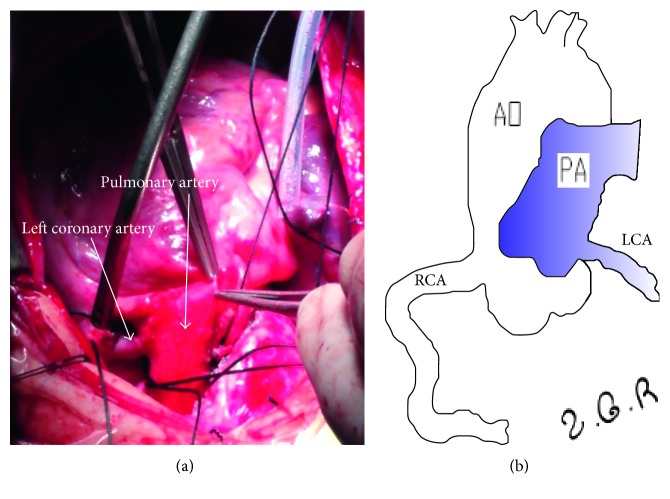
The image in the surgery shows the left coronary artery originating from the pulmonary artery. The diagram displays the right coronary artery (RCA) originating from the aorta artery (AO) while the left coronary artery (LCA) from the pulmonary artery originating from the pulmonary artery (PA).

**Figure 4:**
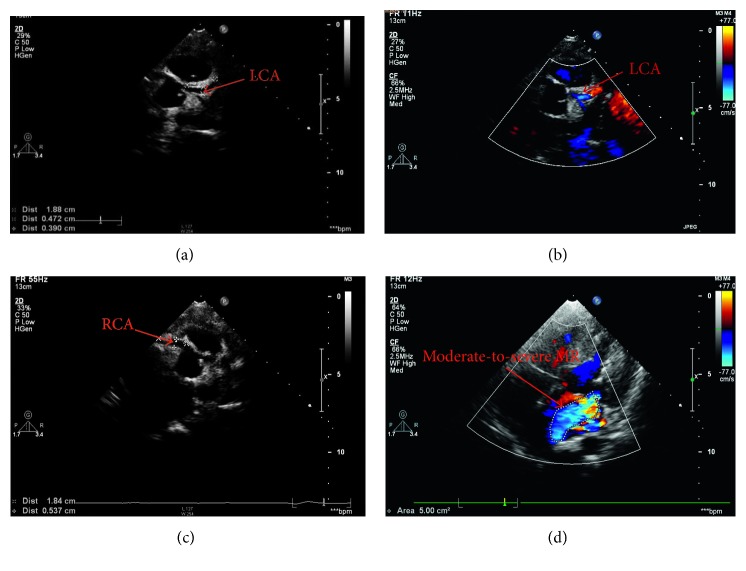
(a, b) The dilated left coronary artery connected to the aorta in the two-dimensional and color doppler flow imaging(CDFI). (c) The dilated right coronary artery connected to the aorta. (d) Moderate-to-severe mitral regurgitation.

**Figure 5:**
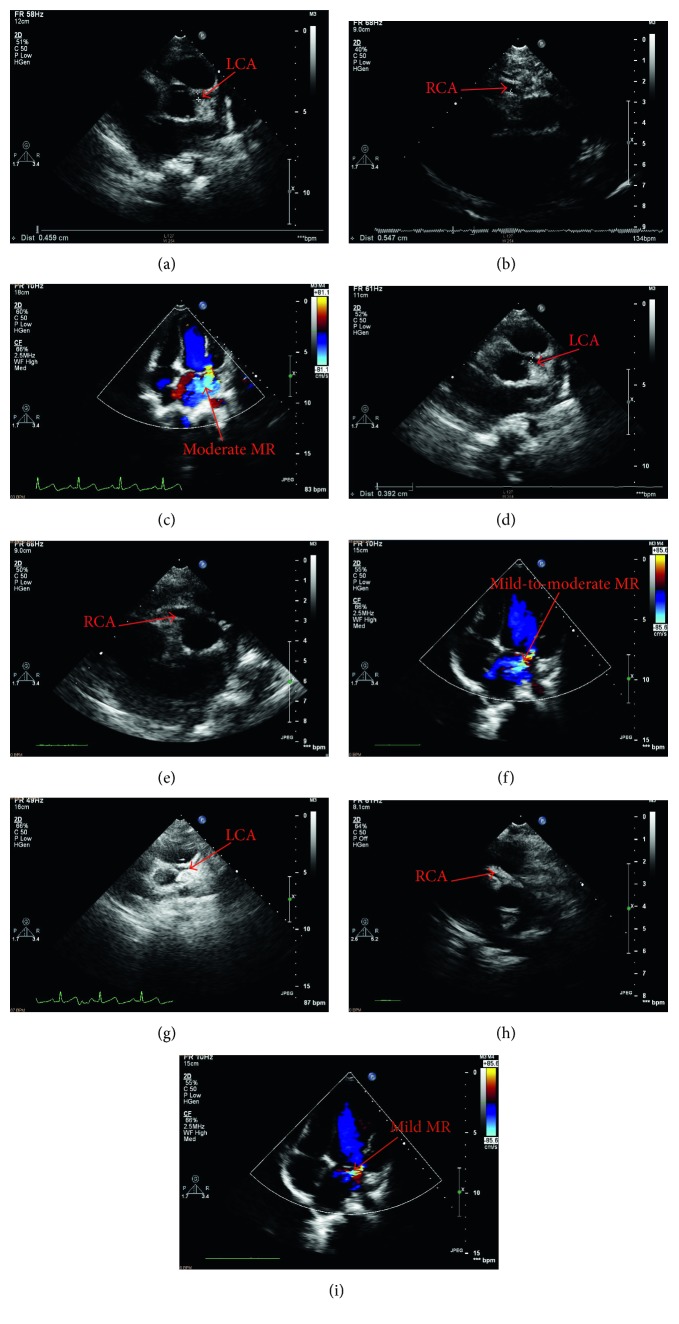
(a, b, c) Bilateral coronary artery aneurysms and moderate mitral regurgitation on the first month after the surgery. (d, e, f) The right coronary artery aneurysms/dilated left coronary artery and mild-to-moderate mitral regurgitation on the sixth month after the surgery. (g, h, i) Bilateral dilated coronary arteries and mild mitral regurgitation on the first year after the surgery.

The patient underwent direct reimplantation of the abnormal LCA into the aorta with general anesthesia under extracorporeal circulation through a median sternotomy. During the procedure, origin of the LCA from the PA trunk, a dilated and tortuous RCA from the RCC, extensive intercoronary collaterals, and anterior mitral valve prolapse were confirmed (Figure 3). Postoperative bedside echocardiography showed LCA connected to aorta sinus, bilateral coronary artery aneurysms (Figure 4), and moderate-to-severe MR with LVEF of 71%, LVFS of 39%. The chest was closed on postoperative day 2, and the patient was discharged on postoperative day 11 with an uneventful recovery. Postoperative chest X-ray showed inflammation and exudation in the lung and mild cardiomegaly. We followed the patient on the first month (bilateral coronary artery aneurysms and moderate MR with LVEF of 61%, LVFS of 32%), sixth month (right coronary artery aneurysms/dilated left coronary artery and mild-to-moderate MR with LVEF of 65%, LVFS of 35%), and first year (bilateral dilated coronary arteries and mild MR with LVEF of 67%, LVFS of 34%) after the surgery by echocardiography (Figure 5), and the patient showed good functional recovery.

## 3. Discussion

ALCAPA is also named Bland–White–Garland syndrome. Clinically, ALCAPA has been divided into adult and infant types according to intercoronary collateral circulation extent. Infant type usually survives only a few months for the reason of myocardial ischemia and heart failure, and only about 10% to 15% of patients survive to adolescence or adulthood [3]. On the other hand, extensive intercoronary collateral circulation usually exists in adult type, forming the “left to right shunt” and “coronary artery steal blood” phenomenon, which could induce to abnormal perfusion of myocardium and sudden death [4]. Our case was defined as the adult type, with relatively abundant collateral circulation and minimal symptoms and a heart murmur.

The incidence of adult type of ALCAPA is very low and often be asymptomatic, so it is usually being misdiagnosed, especially when ECG shows no obvious ST-T change and V waves. Echocardiography is usually the initial diagnostic tool; when the echocardiographer found the imaging feature of the dilation and dysfunction of left ventricular with severe mitral regurgitation, abnormal flow-pattern in the ventricular septum and dilatation of the right coronary artery, and the wide enhancement and thickening of the endocardium, the echocardiographer should not only pay attention to idiopathic endocardial fibroelastosis (EFE) [5] and/or idiopathic dilated cardiomyopathy (DCM) [6] but also should check the origination of the coronary arteries to avoid being misdiagnosed. The typical and direct echocardiographic imaging feature is to show the LCA originating from the pulmonary artery when diagnosing ALCAPA and should also reveal the origin, size, and course of coronary arteries clearly. In addition, the reversal of blood flow in the left coronary artery and abundant collateral circulation are approval to the diagnosis of ALCAPA. Visibly, echocardiography is an indispensable diagnostic tool because of its noninvasive repeatability, low cost, and ability to clearly show the origin of the coronary artery. It can not only determine real-time hemodynamic change, but also can measure heart function in subclinical stage and postoperative follow-up, including the index of heart, resilience, myocardial wall motion, and strain rate [7]. The patient reported in this report was misdiagnosed as having a VSD in the local hospital mainly due to two reasons: first and most importantly, the fistula in the ventricular septum was diagnosed as a VSD; second, the echocardiographer may not have comprehensive knowledge of ALCAPA.

However, DSA with CAG is considered as the gold standard diagnosis of ALCAPA. The use of the contrast agent in severe infant patients remains controversial with regard to ionization radiation and potential allergic reaction [8]. Magnetic resonance angiography (MRA) and computed tomography angiography (CTA) have been increasingly employed for the diagnosis of ALCAPA. CTA is a noninvasive imaging technique with good density and spatial resolution, which has certain advantages to evaluate small blood vessels such as the coronary artery, but it cannot evaluate an intravascular blood flow condition. MRA can be used to diagnose postoperative left ventricular morphology and function in patients with suspected myocardial ischemia; its image quality can be easily influenced by heart rate.

## 4. Conclusion

ALCAPA is a rare congenital heart disease that carries a high mortality rate and may lead to sudden death if left untreated. Typical echocardiographic features and comprehension of ALCAPA are helpful for diagnosis and differential diagnosis. The echocardiography can diagnose ALCAPA accurately and give sufficient information for heart function and operation.
